# Shell-Shock and Suggestion Treatment

**Published:** 1919-03-29

**Authors:** 


### SHELL-SHOCK AND SUGGESTION TREATMENT.

In these days, when the flow of casualties and war sickness into our hospitals has to a large extent ceased, an opportunity occurs for revision of many necessarily hurried medical decisions, and by no means the least urgent are those concerning the war-neuroses and the common so-called shell-shock. It is satisfactory that activity is being displayed in this direction by both the pensions' boards and several large specialised hospitals, reports from whose staffs have already appeared. A large number of cases with relatively slight disability, have been found unfit for military service and returned to their civil occupation with what is popularly considered to be a weakened nervous system. The injury is thought to be slight, yet permanent, and to threaten so strong an influence upon the development of mental characteristics during the remainder of life, that, apart from effect on immediate surroundings and neighbours, the indirect result may be transmitted by heredity to the coming generation.

Left as they are, there is no doubt that a definite possibility exists of detrimental social influence originating from these cases. One knows that there is the widest divergence of opinion as to the best methods of treatment, but we are inclined, at present, to agree with those authorities who claim that in the great majority of instances "shell-shock" has been but a war name for hysteria.

It is no discredit to a soldier, fighting under the terrible conditions of modern war, to have felt fear; in fact very few of those who have passed through its horrors can deny that they did on occasion fear intensely. The effect must vary greatly with the individual, but many have undoubtedly suffered from hysterical perpetuation of the natural results of intense emotion, and of these it is certain that not a few have had no treatment which to any extent allayed their symptoms. Their peace of mind, stability and mental control have improved with rest and care, but the idea of injury and disability has remained. One has but to view the list of cures of complete blindness, various degrees of paralysis, aphonia, etc., to realise the high measure of success resulting from treatment by hypnotic suggestion. The peace-time effects of concussion are too well known for any to deny that with many cases we now meet there may be some actual break in nervous continuity with the natural inhibitory results, but, at the same time, there is a very large number of instances in which the visit to service hospitals of a doctor versed in methods of suggestion treatment has led to the most astonishing improvement in both recent and old-standing cases of shell-shock.

As a rule, there is no question of malingering; the need is that the men should "realise" that they are well and that they have at most to re-educate the non-functioning systems. It seems clear that the greatest advantage to both the State and the patients would result if some system could be devised whereby the thousands of war-neuroses were all again brought under some efficient form of suggestion treatment. We do not venture to predict the percentage of cures which would result, but think there is no doubt it would be considerable.

